# An efficient and robust exfoliated bentonite/Ag_3_PO_4_/AgBr plasmonic photocatalyst for degradation of parabens†

**DOI:** 10.1039/d0ra02455d

**Published:** 2020-04-22

**Authors:** Jianchao Ma, Shurong Yang, Huixian Shi, Jin Pang, Xiaopeng Zhang, Yuxing Wang, Hongqi Sun

**Affiliations:** College of Mining Engineering, Taiyuan University of Technology Taiyuan 030024 Shanxi P. R. China; Institute of New Carbon Materials, Taiyuan University of Technology Taiyuan 030024 Shanxi P. R. China shihuixian@tyut.edu.cn; School of Engineering, Edith Cowan University Joondalup Western Australia 6027 Australia h.sun@ecu.edu.au

## Abstract

Efficient visible-light-driven heterojunction photocatalysts have attracted broad interest owing to their promising adsorption and degradation performances in the removal of organic pollutants. In this study, a mesoporous exfoliated bentonite (EB)/Ag_3_PO_4_/AgBr (30%) photocatalyst was obtained by stripping and exfoliating bentonite as the support for loading Ag_3_PO_4_ and AgBr. The particle size ranges of Ag_3_PO_4_ and AgBr were about 10–30 nm and 5–10 nm, respectively. The exfoliated bentonite could greatly improve the dispersion and adsorption of Ag_3_PO_4_ and AgBr, and significantly enhance the stability of the material during paraben photodegradation. 0.2 g L^−1^ methylparaben (MPB) was completely decomposed over the EB/Ag_3_PO_4_/AgBr (30%) in 40 min under visible light irradiation. In addition, the photocatalytic activity of EB/Ag_3_PO_4_/AgBr (30%) remained at about 91% after five recycling runs manifesting that EB/Ag_3_PO_4_/AgBr (30%) possessed excellent stability. Radical quenching tests revealed that holes (h^+^) and hydroxyl radicals (·OH) were the major radicals. They attacked the side chain on the benzene ring of parabens, which were gradually oxidized to the intermediates, such as benzoic acid, 3-hydroxybenzoic acid, 4-hydroxybenzoic acid, azelaic acid, and eventually became CO_2_ and H_2_O. The enhancement of photocatalytic activity and photo-stability could be ascribed to the stable structural characteristics, enlarged surface area, high absorption ability, and improved light absorption ability from loading Ag_3_PO_4_ onto EB. Meanwhile, the matched energy levels of Ag_3_PO_4_ and AgBr made the photoelectron–hole pairs separate and transfer effectively at the interfaces. As a result, the photocatalytic properties of EB/Ag_3_PO_4_/AgBr (30%) composites were enhanced.

## Abstract 2

A mesoporous exfoliated bentonite (EB)/Ag_3_PO_4_/AgBr (30%) photocatalyst was designed to combine various functions to achieve efficient photodegradation of parabens.

## Graphical abstract

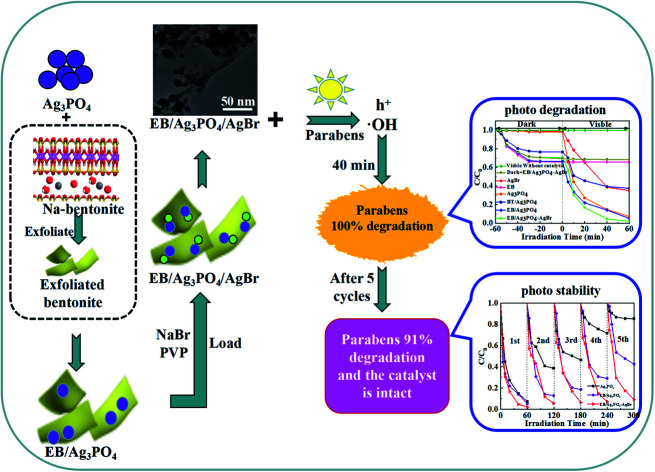

## 1. Introduction

Parabens, such as methylparaben (MPB) and ethylparaben (EPB),^1^ are widely used in food, cosmetics and pharmaceuticals. The effluent from wastewater containing parabens has caused serious environmental problems.^2^ Paraben exposure to the community could induce adverse health issues, such as carcinogenic potential,^3^ endocrine dyscrasia, immune dysfunction and developmental and behavioral disorders.^4^ Thus, the development of efficient treatment technologies has become an urgent need. Many technologies, for example, adsorption,^5^ biodegradation,^6^ and advanced oxidation processes,^7^ have been applied to remove parabens from wastewater. Among advanced oxidation technologies, photocatalysis has been paid much attention because of its energy saving and high efficiency. TiO_2_ is the most commonly used photocatalyst. But it has a large band gap energy (3.2 eV) and cannot respond visible light, which presents 42% solar energy. The limited light absorption of ultraviolet region (5% of solar energy) results in a low removal efficiency in response to sunlight. Coupling TiO_2_ photocatalysis with H_2_O_2_ could result in a higher removal efficiency,^8^ or applying as a self-organized electrode to improve the defects of TiO_2_.^3^ However, the addition of extra oxidants and the design of oxidation electrodes still not extend the light absorption range. The development and exploration of photocatalytic materials with a low band energy is of critical importance. So far, Ag-based catalysts have been widely reported due to their low band gap energies overlying all wavelengths within the near infrared region, showing high photoluminescence quantum yields.

Silver orthophosphate (Ag_3_PO_4_) with an indirect band gap of 2.36 eV has been extensively investigated since it was reported by Ye *et al.*^9^ Although Ag_3_PO_4_ is a prospective photocatalyst for environment remediation,^10^ it has very low solubility in aqueous solution and very easy to occur agglomeration.^11^ These greatly destroy its stability and make it difficult for being recovered after reaction.^12^ It is also prone to photo-corrosion.^13^ Therefore, a cost-effective and efficient material to further improve the stability and reduce the photo-corrosion of Ag_3_PO_4_ can play a critical role for the feasibility.

Recently, many researches aim to search suitable propping materials for increasing dispersion of photocatalyst for enhancing its stability.^14^ Common supports used for photocatalysts are metallic oxide,^15^ ordered mesoporous silica materials,^16^ layered graphite,^17^ hollow glass bead, quartz glass tube (sheet), ordinary (conductive) glass sheet, organic glass,^10^ optical fiber,^1^ natural clay,^14^ activated carbon,^18^ hydrotalcite-like compounds,^19^ and CNTs,^20^*etc.* Clay materials are widely recognized as the good substrates because of their large reserve, low cost, cohesiveness and large specific surface area. Bentonite is abundant on the Earth. It has been extensively served as a native, financial sorbent and upholder for catalysis in sewage disposal.^21^ It possesses remarkable adsorption capacities and owns cation exchange sites at both the interlamination and the outside edges. Thus, bentonite is used to synthesize composite photocatalysts with high activity, such as, ZnFe_2_O_4_/Na–Ben,^21^ HO–Ca–Ben,^22^ BiVO_4_/bentonite,^23^ MoS_2_/bentonite,^24^ ZnS/bentonite.^25^ These composite photocatalysts were effectively used to degrade organic pollutants, because active component of photocatalyst was evenly dispersed on clay matrix producing different reaction sites. Bentonite improved the stability of these catalyst due to a great specific surface area. But it has been widely accepted that the method of interlayer loading is disadvantageous to the photon absorption. So degradation performances of photocatalyst nanoparticles supported on bentonite layers outside are better than that of in the interlayers.^26^ Bentonite was delaminated to form the exfoliation material with the huge external surface area, which overcame the aggregation and instability issues of Ag_3_PO_4_.^27^

The photo-corrosion of Ag_3_PO_4_ still existed after Ag_3_PO_4_ loading on the exfoliated bentonite due to lack of electron receiver.^28^ The exterior was formed by shiny black Ag granules, which would inescapably hinder the sorption of visible light by Ag_3_PO_4_. In recent years, a remarkable way to ameliorate the photocatalytic activity of Ag_3_PO_4_ is to compound it with another semiconductor possessing different band structures, for instance, AgCl,^29^ glass,^10^ WO_3_,^30^ sulfate-doped,^31^ and Fe_2_O_3_,^32^*etc.* The proper alignment of conduction band and valence band position between two outstanding catalysts provides driving force for the separation and transfer of photoinduced electrons and electronic-hole.^33^ AgBr was among the best candidate material for the synthesis of semiconductor heterojunctions because the insoluble AgBr nanoparticles could effectively inhibit the dissolution of Ag_3_PO_4_ in aqueous solutions.^34^ The formation of Ag_3_PO_4_/AgBr heterojunction increased the charge transfer because the excited-electrons by plasma in Ag NPs tend to transfer to AgBr, effectively resisting to the photo-corrosion phenomenon of Ag_3_PO_4_.

Herein, an efficient EB/Ag_3_PO_4_/AgBr (30%) hybrid was synthesized by a nucleation reaction and anion-exchange method. The effect of the complex of AgBr and Ag_3_PO_4_ with different mass fraction on the degradation of MPB was investigated. The structure, morphology, light absorption and photocatalytic activity were studied through a number of characteristic methods. The photochemical degradation ability and stability of EB/Ag_3_PO_4_/AgBr (30%) were investigated using parabens as the degradation substrate. The reactive species trapping experiments and high performance liquid chromatography-mass spectrometry (LC-MS) tests were conducted to propose a possible mechanism. In addition, kinetic studies were conducted.

## 2. Experimental

### 2.1. Materials

Inartificial bentonite was bought from Liaoning, Heishan, China. Sodium bromide (NaBr, 99%), silver nitrate (AgNO_3_, 99%), sodium dihydrogen phosphate (NaH_2_PO_4_·2H_2_O, 99%), methylparaben (C_8_H_8_O_3_, 99%), ethylparaben (C_9_H_10_O_3_, 99%), propylparaben (C_10_H_12_O_3_, 99%), and butylparaben (C_11_H_14_O_3_, 99%) were bought from Guoyao agentia Co., Ltd. All chemicals were of the analytical grade and used without purification.

### 2.2. Exfoliation of bentonite

The double layered of bentonite was peeled adopting a liquid-phase peeling method.^27^ The typical process was as follows: 10 g bentonite was mixed with 200 mL H_2_O dissolved with 0.4 g NaF, then stirred at 80 °C for 90 min. Then, 0.05 g (NaPO_3_)_6_ was added to the suspension which was then transferred into a 250 mL round-bottomed flask to carry out high-speed mechanical agitation and treatment with ultrasound for 60 min, respectively. After ultrasonic oscillation, the mixture was aged for 24 h and afterwards centrifuged with 10 000 rpm for 30 min to segregate colloidal part from the supernatant. The colloidal portion of the supernatant was regarded as the Exfoliated Bentonite (EB).

### 2.3. Preparation of EB/Ag_3_PO_4_ and EB/Ag_3_PO_4_/AgBr

**Fig. 1:**
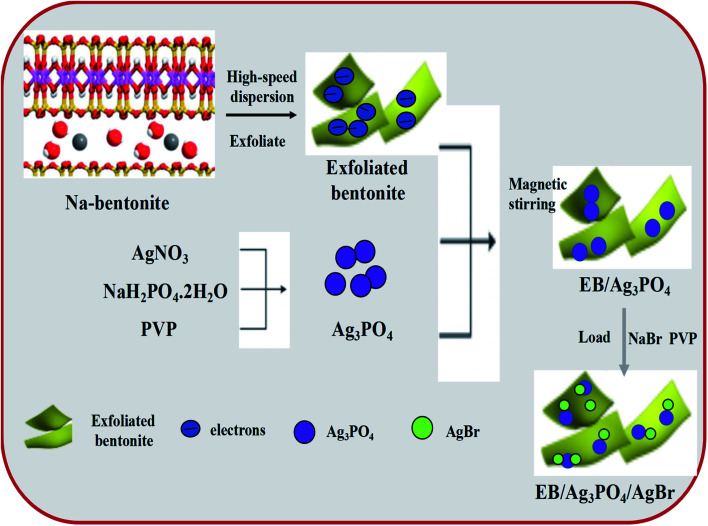
Synthesis procedure for EB/Ag_3_PO_4_/AgBr.

The preparation processes of EB/Ag_3_PO_4_ and EB/Ag_3_PO_4_/AgBr are shown in Fig. 1. EB/Ag_3_PO_4_ was prepared by electrostatic attraction and the nuclear reaction. First, 40 mL aqueous suspension containing 50 g L^−1^ exfoliated bentonite, 0.4 g PVP and 15 mmol AgNO_3_ were mixed, and then 5 mmol NaH_2_PO_4_·2H_2_O was added drop by drop. Ag_3_PO_4_ was precipitated after stirring in darkness for 5 h, and then the olive yellow EB/Ag_3_PO_4_ was gathered by filtration. After rinsing several times with deionized water, EB/Ag_3_PO_4_ nanoparticles were obtained after vacuum drying at 60 °C for 12 h. Meanwhile, 0.84 g EB/Ag_3_PO_4_ nanoparticles and 0.1 g PVP were dispersed in 50 mL of water by ultrasound. After that 0.025 mol L^−1^ NaBr solution was rapidly dropped into the mixture under the magnetic stirring at dark for 120 min at room temperature. And then the EB/Ag_3_PO_4_/AgBr photocatalysts were prepared after they were collected by vacuum drying at 60 °C for 12 h. The series of photocatalysts prepared were labeled as EB/Ag_3_PO_4_/AgBr (*X*) (*X* represents the mass fraction of AgBr in the composite system. *X* = 10%, 20%, 30%, 40%, and 50%), and the composite (Fig. S1†) with 30% AgBr was evaluated to be the best catalyst for the subsequent experiments.

### 2.4. Characterization

The photocatalysts were characterized with XRD patterns obtained with a DX-2700 diffraction instrument. The appearance images were taken on an emission scanning electron microscope (JSM-7800F). TEM was conducted using a JEOL JEM-2010 equipment. UV-Vis spectra were obtained from the Lambda 950 equipment. X-ray photoelectron spectroscopy (XPS) spectra were acquired using monochromatic Al-Kα radiation on a Thermo K-Alpha device. Specific surface areas, and aperture of the compounds were analyzed using the Micromeritics Instruments (ASAP 2010). MPB and the degradation pathway was also explored using LC-MS, on a PerkinElmer Clarus 500 system gas chromatography equipped with a quadrupole spectrometer.

### 2.5. Photocatalytic MPB degradation

The degradation performances of the materials were evaluated by the photocatalytic oxidation of parabens. MPB was the target pollutant, and degradation performance of other parabens was also discussed. 100 mL MPB solutions (20 mg L^−1^) and 0.1 g catalyst were stirred in dark for 1 h to guarantee adsorption–desorption equilibrium. The reaction system was then put under a 300 W Xe arc lamp. At given interval of time, collecting and centrifuging of 4 mL suspension. The centrifugal fluid was injected into a colorimetric dish by a 0.22 μm filter a syringe. The degradation performance of parabens was measured in UV-vis spectra at 308 nm (256 nm). The percentage removal could be calculated by eqn (1):

1 *C*/*C*_0_ = (*C*_0_ − *C*)/*C*_0_ × 100%

*C*_0_ is the primeval consistence of paraben (mg L^−1^) and *C* is the consistence of MPB after a certain amount of illumination time *t* (min).

The stability of composites was evaluated as below: at every run of cyclic experiment, the suspension was extracted and abandoned. The amount of reaction substrates was measured by 20% loss of catalyst per time.

## 3. Results and discussion

### 3.1. Characterization results

**Fig. 2:**
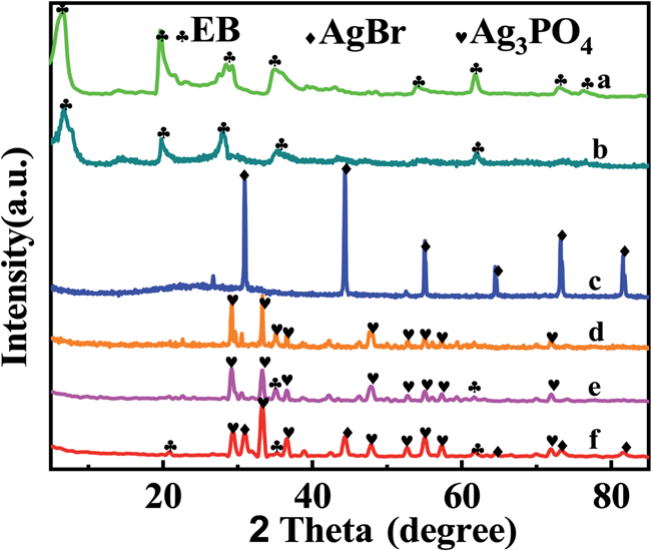
XRD patterns of (a) natural bentonite, (b) EB, (c) AgBr, (d) Ag_3_PO_4_, (e) EB/Ag_3_PO_4_, (f) EB/Ag_3_PO_4_/AgBr (30%).

Fig. 2(a) displays the XRD patterns. The peaks at 2*θ* = 6.84°, 20.12°, and 28.7° are from natural bentonite (JCPDS no. 79-1910). The exfoliation bentonite was obtained and confirmed by the broad and weak peak at a low angle (2*θ* < 10°), as shown in Fig. 2(b). Ag_3_PO_4_ (JCPDS no. 06-0505)^35^ and AgBr (JCPDS no. 06-0438)^36^ with high crystallinity can be observed in Fig. 2(c) and (d), respectively. They played the main photocatalytic roles in the composite photocatalyst. Fig. 2(e) shows the characteristic peak of EB and Ag_3_PO_4_, revealing the synthesis of EB/Ag_3_PO_4_ composites. The peak reduction of the exfoliated bentonite may be as a result of the damage of bentonite gel when it was washed. From Fig. 2(f), AgBr, EB and Ag_3_PO_4_ are found, indicating that EB/Ag_3_PO_4_/AgBr (30%) composites were successful synthesized. In addition, from the XRD picture, AgBr did not alter the peak locations of Ag_3_PO_4_, showing that AgBr was loaded on the surface of Ag_3_PO_4_.

**Fig. 3:**
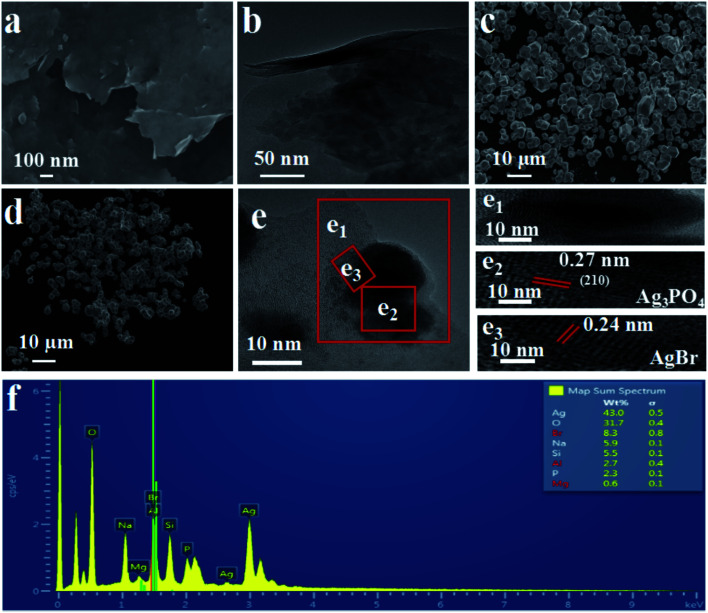
SEM images of (a) EB and HRTEM images of (b) EB, SEM images of (c) Ag_3_PO_4_, (d) AgBr, (e) HRTEM images of EB/Ag_3_PO_4_/AgBr (30%), and (f) the corresponding EDS pattern for EB/Ag_3_PO_4_/AgBr (30%) composite.

The SEM (Fig. 3(a)) and HRTEM (Fig. 3(b)) images of exfoliated bentonite are exhibited in Fig. 3. The exfoliated bentonite was like a “cicada's” wing and possesses smooth, thin-layered structures with curly edges, indicating that the bilayer bentonite has been stratified.

Fig. 3(c) presents that Ag_3_PO_4_ is of an irregular rugby ball morphology while AgBr is of a regular spherical shape, as shown in Fig. 3(d). HRTEM image in Fig. 3(e) shows that the granule size range of Ag_3_PO_4_ and AgBr is about 10–30 and 5–10 nm, respectively. And there was direct alignment between the EB/Ag_3_PO_4_ and AgBr in this heterojunction. Thus, EB/Ag_3_PO_4_/AgBr (30%) was favorable for surface charge transfer. In the HRTEM images, two different kinds of lattice fringes can be observed. One of the fringe intervals is 0.24 nm, which may match the interplanar spacing of plane for AgBr. The other one is 0.27 nm, which could be assigned to the (210) crystallographic plane of Ag_3_PO_4_.^37^ It can then be deduced that the heterojunction structure was formed between the two phases. Based on the above results, the hybrid of EB/Ag_3_PO_4_/AgBr (30%) with a stable structure was confirmed. This revealed that the formation of hybrid of EB/Ag_3_PO_4_/AgBr (30%) could be conducive to the transfer of photoinduced electron–hole pairs and enhance the photoactivity, which is beneficial to the degradation of organic pollutants.

In addition, Fig. 3(f) shows the EDS mapping of the EB/Ag_3_PO_4_/AgBr (30%). O, P and Ag peaks were observed, where the content of Ag reaches 43%. Br signals corresponding to AgBr were also observed, further confirming that AgBr was produced on the outside surface of Ag_3_PO_4_ by ion-exchange reaction. Besides, Na, Si, Al, Mg were homogeneously distributed in the EB/Ag_3_PO_4_/AgBr (30%) particles, attributing to the exfoliated bentonite. These further proved that EB/Ag_3_PO_4_/AgBr (30%) was successfully prepared.

**Fig. 4:**
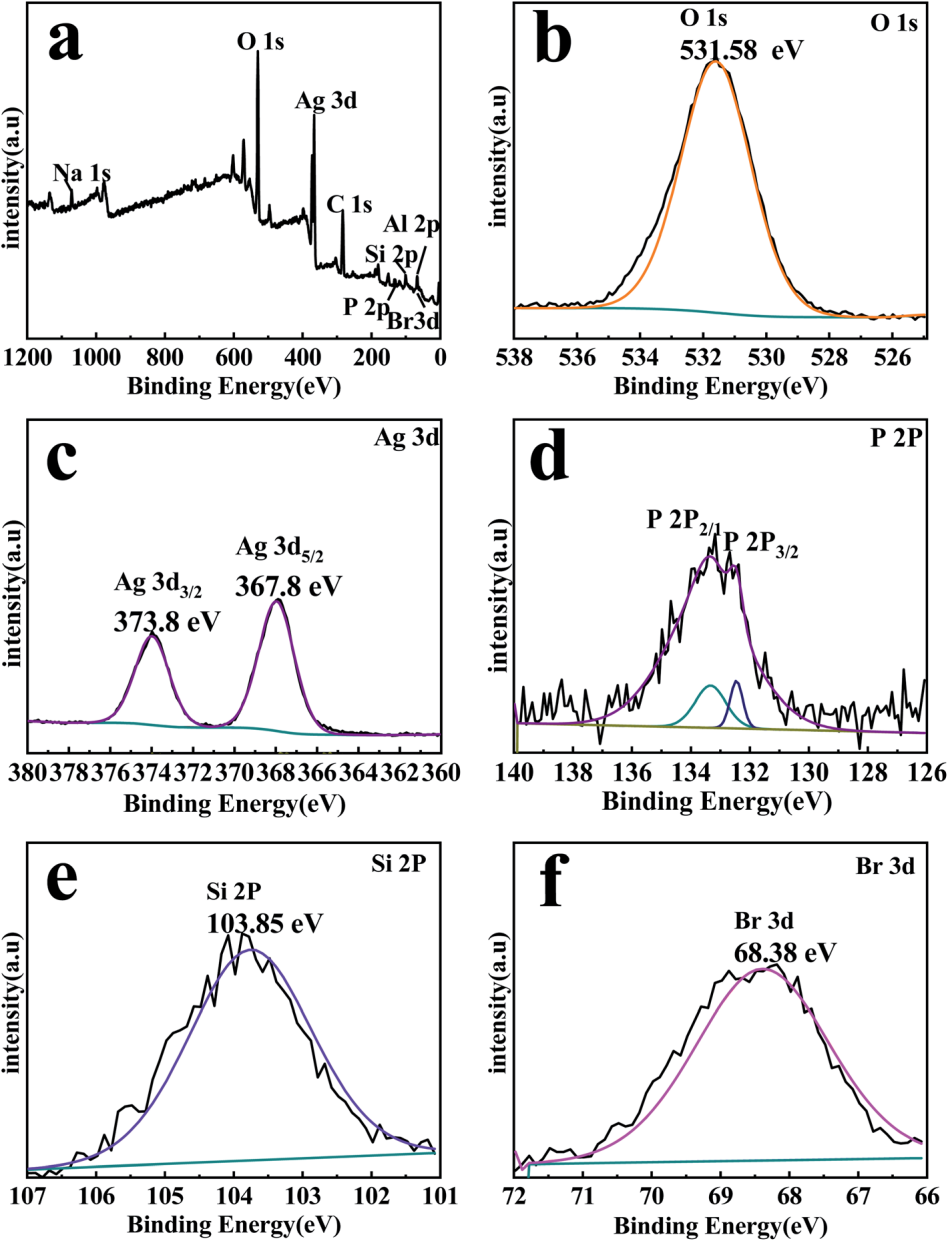
XPS spectra of EB/Ag_3_PO_4_/AgBr (30%) compound: (a) full-spectrum, (b) O 1s and (c) Ag 3d, (d) P 2p, (e) Si 2p, and (f) Br 3d.

The compositions and element valence states of the EB/Ag_3_PO_4_/AgBr (30%) were detected through XPS, the results are shown in Fig. 4. The XPS spectrum (Fig. 4(a)) displays C 1s, O 1s, Ag 3d, Br 3d and P 2p, Si 2p, Al 2p, Na 1s peaks for EB/Ag_3_PO_4_/AgBr (30%), which are consistent with the chemical compositions of the photocatalyst. The peak at 531.58 eV in Fig. 4(b) originating from the lattice oxygen of Ag_3_PO_4_.^29^ Two individual peaks of the Ag 3d spectra in Fig. 4(c) are at about 367.8 and 373.8 eV, corresponding to Ag 3d_5_/_2_ and Ag 3d_3_/_2_, separately, they were ascribed to Ag^+^ from Ag_3_PO_4_ and AgBr.^17^ No XPS peaks of Ag nanocrystals were found, indicating that Ag^o^ could not be formed during the preparation procedure. Two peaks of P 2p at 133.2 and 132.52 eV were fully justified of P 2p_1_/_2_ and P 2p_3_/_2_, separately,^29^ as appeared in Fig. 4(d). In addition, The Si 2p peak at 103.85 eV is corresponding to SiO_2_ (Fig. 4(e)), which is the main component of exfoliated bentonite.^21^ The exfoliated bentonite would provide a larger contact area for photocatalytic reaction. It could also promote photo-induced carriers' separation between Ag_3_PO_4_ and AgBr on the EB/Ag_3_PO_4_/AgBr (30%), and greatly enhance the stability of the photocatalyst. The binding energy of Br 3d was 68.38 eV in Fig. 4(f), which could be ascribed to the lattice of Br^−^ in AgBr.^38^

**Fig. 5:**
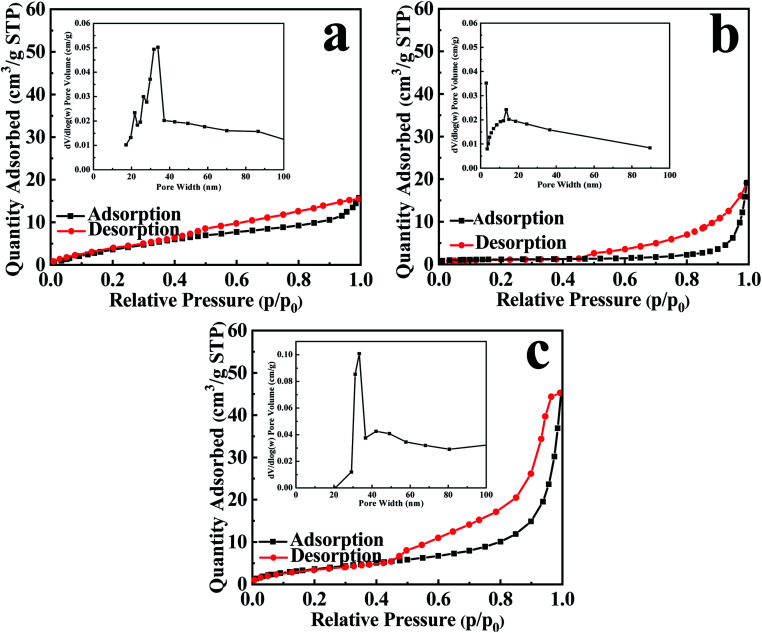
N_2_ adsorption–desorption isothermal curve and pore size distribution of (a) EB, (b) EB/Ag_3_PO_4_, and (c) EB/Ag_3_PO_4_/AgBr (30%).

Fig. 5 presents the adsorption isotherm of stripped-bentonite belongs to type II isotherms, suggesting non-porous or macroporous solid materials. Fig. 5(b) and (c) show that EB/Ag_3_PO_4_ and EB/Ag_3_PO_4_/AgBr (30%) are mesoporous materials. The inset of Fig. 5 shows that EB/Ag_3_PO_4_ and EB/Ag_3_PO_4_/AgBr (30%) had average pore sizes of 13.53 and 33.3 nm, respectively. The pore size distributions of both catalysts exhibited the narrow mesoporous range, showing that they had the regular mesoporous framework. Table S1† suggests that the pore capacity and aperture of EB/Ag_3_PO_4_/AgBr (30%) were significantly larger than EB/Ag_3_PO_4_ and stripped-bentonite. The larger pore size for EB/Ag_3_PO_4_/AgBr (30%) than ones for exfoliated-bentonite and EB/Ag_3_PO_4_ may be attributed to the AgBr solidifying on the pore walls and suppressing the structure collapse of the microporous stripped-bentonite and the mesoporous EB/Ag_3_PO_4_. Compared with the exfoliated bentonite, the reasons for the fractionally diminish in the specific surface area of EB/Ag_3_PO_4_ and EB/Ag_3_PO_4_/AgBr (30%) are Ag_3_PO_4_ or both Ag_3_PO_4_ and AgBr support on the exfoliated bentonite. It could be explained that EB containing Ag_3_PO_4_ and AgBr formed three-dimensional mesoporous EB/Ag_3_PO_4_/AgBr (30%) which can greatly improve the stability of EB/Ag_3_PO_4_/AgBr (30%). Moreover, a large specific surface area of exfoliated bentonite could solve agglomeration of Ag_3_PO_4_ and poor dispersion of heterojunction formed of Ag_3_PO_4_ and AgBr.

**Fig. 6:**
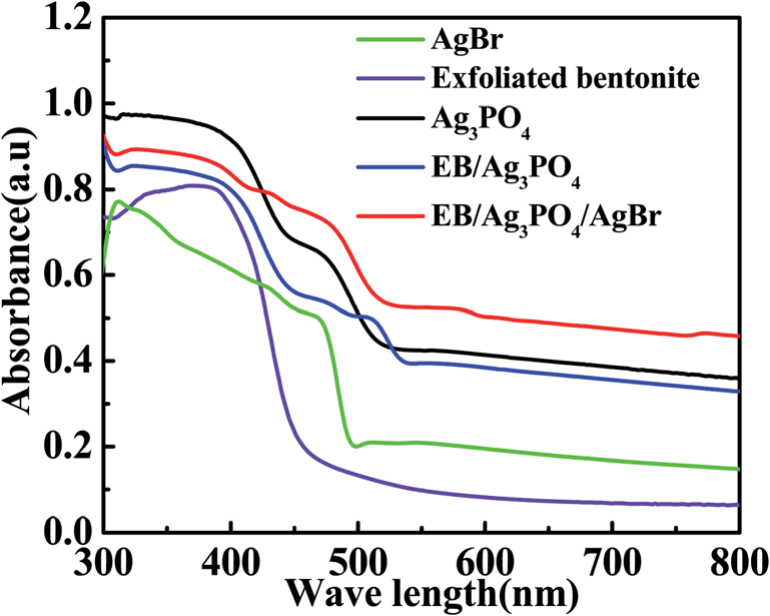
UV-vis diffuses reflectance absorption spectra of AgBr, EB, pure Ag_3_PO_4_, EB/Ag_3_PO_4_ and EB/Ag_3_PO_4_/AgBr (30%).

The UV-vis spectra of the designed catalysts are shown in Fig. 6. AgBr has a photoabsorption edge is about 481 nm.^33^ Ag_3_PO_4_ has a wide photoabsorption region, and the light absorption distribution from UV light region to 530 nm,^9^ echoing the band gap (E_g_) of 2.36 ev. The exfoliated bentonite hardly absorbs the visible light. But the EB/Ag_3_PO_4_ emerged a mixed absorbance of EB and Ag_3_PO_4_ in comparison with the bare Ag_3_PO_4_, existing a slight band redshift. When AgBr was introduced into EB/Ag_3_PO_4_ to form the heterojunction structure EB/Ag_3_PO_4_/AgBr (30%), the absorption band of 400–800 nm and stronger light response were observed in the entire visible light region. This could reveal that the strong interaction between the monomers of the composite allowed the solar spectrum to be used more effectively. Moreover, the local surface plasmon response of Ag and the double heterojunctions of Ag_3_PO_4_ and AgBr could improve the efficiency of photocharge transfer and inhibit the charge recombination, which enhanced the photocatalytic property of EB/Ag_3_PO_4_/AgBr (30%).

To investigate the separation of photogenerated electron–hole pairs more deeply, photocurrent responses of the Ag_3_PO_4_, and EB/Ag_3_PO_4_/AgBr (30%) composites samples were recorded and the results are shown in Fig. S2.†^12^ The photocurrent response of EB/Ag_3_PO_4_/AgBr (30%) electrodes under visible light irradiations was observed. However, the photocurrent of Ag_3_PO_4_ was not detected under the same test conditions. It can be found that EB/Ag_3_PO_4_/AgBr (30%) exhibited a constant photocurrent density during 20 min light irradiation, indicating a higher photoelectric conversion efficiency and higher stability of EB/Ag_3_PO_4_/AgBr (30%) than pristine Ag_3_PO_4_. It can be inferred that the heterojunction structure formed in the composite is conducive to the separation of electrons and holes due to the matched energy level structure and the interface interaction between Ag_3_PO_4_ and AgBr loaded on the exfoliated bentonite. Therefore, a stronger photocurrent than the active component Ag_3_PO_4_ is produced.^39^ This will make the composite have a stronger degradation effect on pollutants.

### 3.2. Photocatalytic activity

**Fig. 7:**
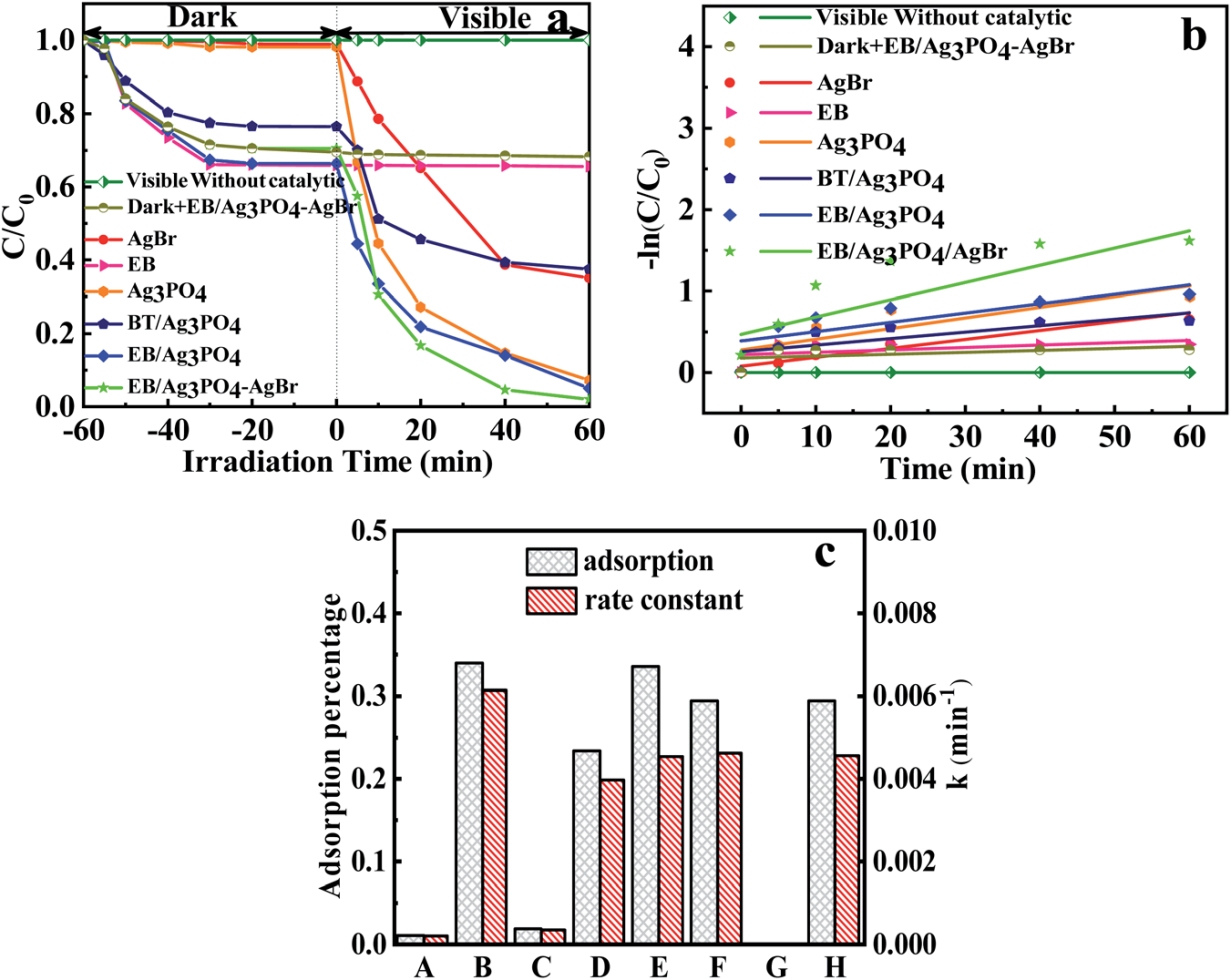
(a) Photocatalytic degradation of MPB by composites; (b) kinetics fitting for the degradation of MPB; (c) adsorption properties and rate coefficients of (A) AgBr, (B) exfoliated bentonite, (C) Ag_3_PO_4_, (D) bentonite/Ag_3_PO_4_ composites, (E) EB/Ag_3_PO_4_, (F) EB/Ag_3_PO_4_/AgBr (30%), (G) visible without catalytic, (H) dark + EB/Ag_3_PO_4_/AgBr (30%).

From Fig. 7(a), the adsorption–desorption equilibrium of MPB was reached in 60 min. Thus, the photocatalytic degradation react could be commenced under light after 60 min, as shown in eqn (2).

2 Ag_3_PO_4_/AgBr + light → Ag_3_PO_4_ (e^−^ + h^+^)/AgBr (e^−^ + h^+^)

The line of a blank experiment (under visible light without a catalyst) in Fig. 7(a) was stable, showing the negligible self-photolysis of MPB without photocatalysis under visible light irradiations.^40^ Addition of EB has only adsorption effect on MPB. Moreover, the MPB degradation reached 64.97% and 92.79% with the addition of AgBr and Ag_3_PO_4_, respectively. But only 62.47% of MPB was degraded with BT/Ag_3_PO_4_. It seemed that Ag_3_PO_4_ was inserted into interlayer and decreased its activity. In comparison, EB/Ag_3_PO_4_ possessed a good activity (94.99%). The introduction of AgBr into EB/Ag_3_PO_4_ enhanced the photocatalytic activity, whereas MPB completely reached degradation in 40 min. Compared with AgBr, Ag_3_PO_4_, EB and BT/Ag_3_PO_4_, EB/Ag_3_PO_4_, respectively, EB/Ag_3_PO_4_/AgBr (30%) unfolded the supreme catalytic function. This can be credited to loading Ag_3_PO_4_ on the exfoliated bentonite, which improved the dispersion of Ag_3_PO_4_ and enhanced the stability of EB/Ag_3_PO_4_/AgBr (30%). Introduction of AgBr into EB/Ag_3_PO_4_ could greatly enhance the catalytic function owing to the retardation of the light corrosion about Ag_3_PO_4_. Fig. S3 and S4† show that the degradation rate of EPB, PPB, and BuPB is 99.4%, 95.6%, and 93.93%, respectively. The results confirmed that EB/Ag_3_PO_4_/AgBr (30%) has strong visible light catalytic properties for the decomposition of paraben. The reaction rate constant of EB/Ag_3_PO_4_/AgBr (30%) (*K*_3_ = 0.0358 min^−1^) for MPB degradation was more than 4 times of that on Ag_3_PO_4_ (*K*_1_ = 0.011 min^−1^), and more than 2.75 times of that on EB/Ag_3_PO_4_ (*K*_2_ = 0.013 min^−1^). It suggests that the modification of Ag_3_PO_4_ with EB and AgBr can significantly improve the photocatalytic properties of materials under the irradiations.

Fig. 7(c) reveals that the adsorption capacity before sunlight irradiations for AgBr, EB, Ag_3_PO_4_, BT/Ag_3_PO_4_, EB/Ag_3_PO_4_ and EB/Ag_3_PO_4_/AgBr (30%) are 1.092%, 33.97%, 1.88%, 23.4%, 33.57% and 29.45%, respectively. EB displays a distinctly higher absorptivity for Ag_3_PO_4_, which was beneficial for strengthening the photochemical catalysis function and stability. Adsorption capacity of EB/Ag_3_PO_4_/AgBr (30%) is lower than EB/Ag_3_PO_4_ although the reaction rate was much faster. Noteworthy, EB/Ag_3_PO_4_/AgBr (30%) presented the maximum reaction rate constant, which was approximately 13 times and 1.1 times more than that of the Ag_3_PO_4_ and EB/Ag_3_PO_4_, respectively. This could effectively reduce the overall degradation time of MPB to achieve rapid degradation of MPB in industrial wastewater treatment. This also indicated that the adsorption capability was the one of reasons for the high degradation activity of EB/Ag_3_PO_4_/AgBr (30%).

### 3.3. Reusability and stability of the photocatalysts

**Fig. 8:**
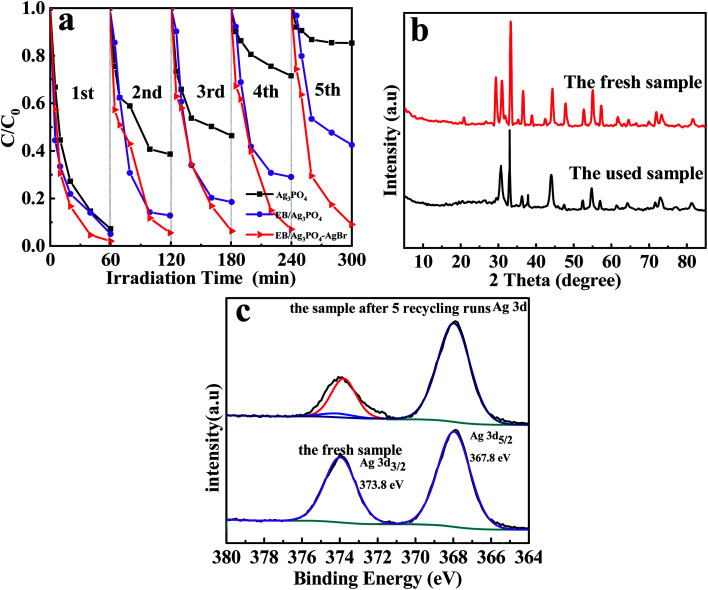
(a) Recycling experiments for MBP photodegradation by Ag_3_PO_4_, EB/Ag_3_PO_4_ and EB/Ag_3_PO_4_/AgBr (30%) under the illumination, (b) the XRD patterns of EB/Ag_3_PO_4_/AgBr (30%) after 5 cycle experiments, and (c) the XPS patterns of EB/Ag_3_PO_4_/AgBr (30%) after 5 cycle experiments.

Recycling experiments of MPB degradation over EB/Ag_3_PO_4_/AgBr (30%) were implemented to assess the stability of materials. From Fig. 8(a), the MPB decomposition on Ag_3_PO_4_ and EB/Ag_3_PO_4_ decreased to 14% and 57% after 5 consecutive cycles, respectively. By contrary, the degradation rate of EB/Ag_3_PO_4_/AgBr (30%) to MPB was still 91% after five recycling runs, revealing that it had excellent stability under the illumination. To further unveil the stability of catalyst, XRD was tested to compare the crystal structure changes of the EB/Ag_3_PO_4_/AgBr (30%) before and after the reaction. Fig. 8(b) shows that the utilized EB/Ag_3_PO_4_/AgBr (30%) had a similar crystal structure with the fresh one. No additional characteristic peaks were observed in the used EB/Ag_3_PO_4_/AgBr (30%), implying that no crystalline structure was evidently changed after the photocatalytic reactions.

The used EB/Ag_3_PO_4_/AgBr (30%) was also characterized by XPS, and giving the outcomes as follows in Fig. 8(c). After 5 cycle experiments, the binding energies of Ag 3d_5_/_2_ and Ag 3d_3_/_2_ are 367.89 and 373.99 eV about the used EB/Ag_3_PO_4_/AgBr (30%), respectively. The Ag 3d_3_/_2_ was fitted into two distinct peaks (373.8 and 374.15 eV).^41^ The values of Ag 3d at 373.8 and 367.89 eV echoed Ag^+^.^42^ The peak at 374.15 eV was due to metallic silver, which was much smaller than ones of Ag^+^.^43^ The formation of a small amount of Ag^o^ would not wrap Ag_3_PO_4_ to affect the activity of the active component but form a plasma effect to boost the photocatalytic oxidation of the samples. The intensity of Ag^+^ decreases and Ag^o^ increases suggested that EB/Ag_3_PO_4_/AgBr (30%) was turned into EB/Ag_3_PO_4_/AgBr (30%)@Ag in the procedure of photochemical catalysis. It could form a stable plasma heterojunction photocatalyst because of the formation of Ag^o^ with plasma resonance effect between Ag_3_PO_4_ and AgBr. The results further demonstrated that AgBr and Ag_3_PO_4_ supported by exfoliated bentonite could significantly increase the photocatalytic degradation stability and activity.

### 3.4. Mechanistic studies

**Fig. 9:**
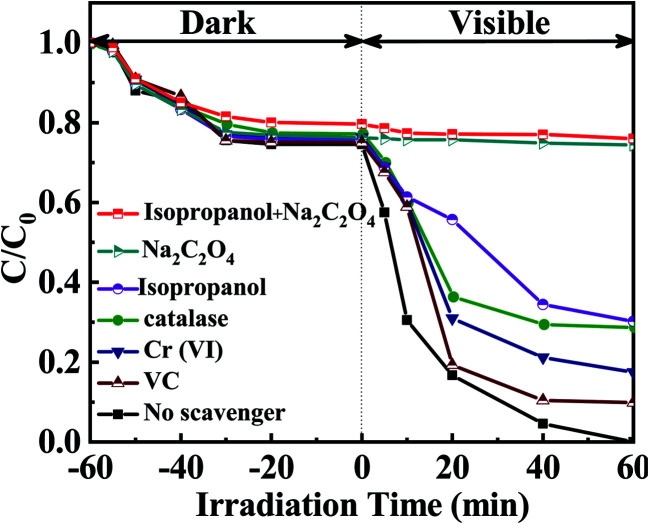
Effect of different quenching agents on the decomposition of MPB over EB/Ag_3_PO_4_/AgBr (30%) under irradiations.

It has been widely accepted that the photoinduced holes (h^+^), ·O_2_^−^, and ·OH radicals^44^ are the several reactive substances in the photocatalytic process.^45^ Thus, catalase, Cr(vi), Na_2_C_2_O_4_, isopropanol^46^ and vitamin C (VC) were used to investigate the contribution rate of H_2_O_2_,^47^ e^−^, h^+^, ·OH, and ·O_2_^−^ (ref. 20) for MPB degradation. The results are exhibited in Fig. 9. The removal of MPB decreased to 70.92% with the addition of catalase. The contribution of H_2_O_2_ was 29.08%, improving that H_2_O_2_ acted a certain part during the process of photochemical catalysis deactivation. Degradation rate of MPB dropped to 90.11% or 82.55% with the introduction of VC or Cr(vi), respectively. These implied that ·O_2_^−^ and e^−^ contributed 9.89% and 17.45% to the reduction of MPB. Interestingly, degradation rate of MPB reached 25.59% and displayed a significant inhibitory effect with the addition of Na_2_C_2_O_4_, demonstrating that the contribution of h^+^ to removal rate of MPB was 74.41%. The results indicated that the holes were the most important component in the photolysis of MPB. When isopropanol was added, ·OH^48^ contributed 30.88% to the degradation efficiency of MPB, revealing that ·OH played the second major role in the decomposition of MPB. Besides, to the photocatalytic process, the combination of Na_2_C_2_O_4_ and isopropanol were employed to go a step further verify the dedications of h^+^ and ·OH, and the degradation degree of MPB was 24%. Nevertheless, the addition of VC, Cr(vi) and catalase at the same time had a feeble inhibitory effect on the removal of MPB. Therefore, it concludes that all the h^+^ and ·OH contributed to the high photo-catalytic properties of EB/Ag_3_PO_4_/AgBr (30%) for the removal of MPB.

In order to further explore the possible degradation pathway, LC-MS analysis was carried out on the products of MPB photodegradation over EB/Ag_3_PO_4_/AgBr (30%), and the outcomes are presented in the Fig. S5.† The main photodegradation intermediates of MPB are hydroxylated substances including benzoic acid, 3-hydroxybenzoic acid, 4-hydroxybenzoic acid and other benzene substances. These hydroxylated products can be further oxidized and discomposed by the attack of h^+^ and ·OH. Benzoic acid was formed by the oxidation decomposition of the side chain of benzene ring. Along with the oxygenolysis further reacts, benzoic substances such as 3-hydroxybenzoic acid and 4-hydroxybenzoic acid were obtained by the further degradation of benzoic acid. With the reaction proceeding, the above products could be oxidized and generate some smaller molecule carboxylic acids before the formation of carbon dioxide and water.

**Fig. 10:**
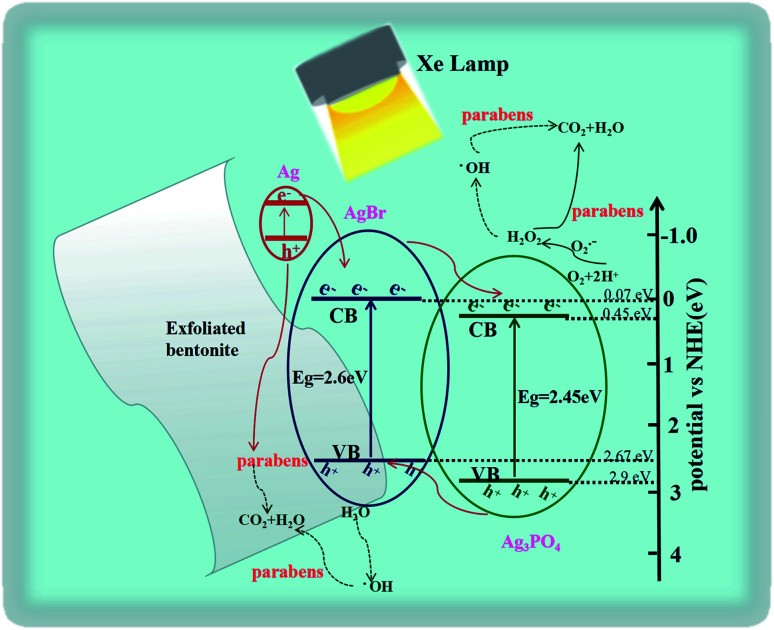
The photodegradation mechanism of MPB on EB/Ag_3_PO_4_/AgBr (30%).

Based on the above outcomes, the feasible mechanism of photodecomposition of parabens on EB/Ag_3_PO_4_/AgBr (30%) was proposed. The light adsorption capacity of EB/Ag_3_PO_4_/AgBr (30%) in the visible region was improved (Fig. 10). The work function about AgBr was higher than that of Ag (*∅*_Ag_ = 4.25 eV, *∅*_AgBr_ = 5.3 eV) while Fermi energy level was lower. Thus, electrons were shifted directly from the plasma metal Ag to the guide band of AgBr. The energy of incident photon was converted by Ag and entered the local surface plasmon resonance (SPR) vibration^49^ and transfers its energy to Ag_3_PO_4_ by resonance energy transfer, generating electron–hole pairs in Ag_3_PO_4_. Thus, SPR electrons generated by Ag and photogenerated electrons obtained by AgBr could be readily diverted to the interface of Ag_3_PO_4_, which greatly enhances the photocatalyst property in the light.

It was well known that effective suppression of electron–hole pair recombination was essential to improve photocatalytic activity.^42^ Under the illumination, an animate photon rapidly excited the electron transition through the Ag_3_PO_4_ and AgBr valence band (VB) to the conduction band (CB) (eqn (2)).^40^ The photosensitive electrons on the CB of AgBr could shift liberally to the CB of Ag_3_PO_4_, while the h^+^ on the VB of Ag_3_PO_4_ could freely move to the VB of AgBr. These lead to that the recombination of electron–hole pairs was effectively suppressed (eqn (3)). AgBr (CB = 0.07 eV, VB = 2.67 eV) had minus conduction and valence band potentials than Ag_3_PO_4_ (CB = 0.45 eV, VB = 2.90 eV).^27^ Thus, SPR electrons generated by Ag and photoinduced electrons generated by AgBr could be readily diverted to the interface of Ag_3_PO_4._ The photogenerated holes of Ag_3_PO_4_ could be also migrated on the outer face of AgBr. This could powerfully restrain the charge re-merge, making more carriers ready for the decomposition of parabens.^43^ Plasma electrons would be ejected into the conduction band of Ag_3_PO_4_, which could be captured and reacted with O_2_ to form ·O_2_^−^ (eqn (4)). Furthermore, ·O_2_^−^ and H_2_O might be transformed into H_2_O_2_ and ·OH to oxidize parabens (eqn (5)). The resulting active substances could be rapidly used to decompose organic matter, moreover, the holes produced by Ag^o^ would have a great synergistic effect for parabens oxidization.^44^ Concurrently, the holes generated by Ag_3_PO_4_ move toward AgBr, and further participate in the degradation reaction of the organics (eqn (6)). In addition, the holes have the interaction with H_2_O to produce ·OH (eqn (7)) for parabens decomposition. Our work suggested that the mechanism for the photodegradation of MPB over EB/Ag_3_PO_4_/AgBr (30%) can be explained as follows: (a) the plasma metal Ag can efficiently shift the photogenerated electrons to Ag_3_PO_4_ and AgBr for reacting with O_2_ to form ·O_2_^−^, H_2_O_2_ and ·OH to oxidize parabens. (b) The holes produced by Ag^o^ would also act on the oxidation of parabens. (c) Photoinduced h^+^ and ·OH radicals directly produced by the h^+^ can lead to the mineralization of organic pollutants into CO_2_ and H_2_O.

3 Ag_3_PO_4_ (e^−^ + h^+^)/AgBr (e^−^ + h^+^) → AgBr (h^+^)/Ag_3_PO_4_ (e^−^)

4 O_2_ + Ag_3_PO_4_ (e^−^) → ·O_2_^−^

5 ·O_2_^−^ + H_2_O → ·OH + H_2_O_2_

6 AgBr (h^+^) → CO_2_ + H_2_O

7 AgBr (h^+^) + H_2_O → ·OH

Therefore, high activity and stability of EB/Ag_3_PO_4_/AgBr (30%) can be explained as follows. Structural stability of EB/Ag_3_PO_4_/AgBr (30%) composite can be greatly enhanced because of Ag_3_PO_4_ loaded on the surface of exfoliated bentonite. Besides, EB/Ag_3_PO_4_/AgBr (30%) exhibited a large BET external surface area and kept a good adsorption performance of organic pollutants molecules. Furthermore, bulk heterojunction of AgBr and Ag_3_PO_4_ realized *in situ* photo-reduced Ag nanoparticle, which prominently diminished the association rate of electron–hole pairs on EB/Ag_3_PO_4_/AgBr (30%) and improved photocatalytic activity.

## 4. Conclusions

EB/Ag_3_PO_4_/AgBr (30%) hybrid was successfully synthesized using a deposition–precipitation and *in situ* anion-exchange methods. EB/Ag_3_PO_4_/AgBr (30%) exhibited superior photocatalytic performance to pure Ag_3_PO_4_ and EB/Ag_3_PO_4_ in the parabens photodegradation under visible light irradiations. Furthermore, degradation efficiency of MPB still remained 91% after 5 recycled tests. Degradation of MPB was mainly *via* ·OH and h^+^ oxidation decomposition over EB/Ag_3_PO_4_/AgBr (30%). The high activity and stability of EB/Ag_3_PO_4_/AgBr (30%) can be attributed to the strong structural stability of materials, which significantly reduced the recombination rate of the electron–hole pairs.

## Conflicts of interest

There are no conflicts to declare.

## Supplementary Material

RA-010-D0RA02455D-s001
