# Supplementary material for: An efficient and robust exfoliated bentonite/Ag3PO4/AgBr plasmonic photocatalyst for degradation of parabens
Source: RSC Adv. 2020 Apr 22;10(27):16027–37. doi: 10.1039/d0ra02455d (PMC9052784; doi:10.1039/d0ra02455d)
Supplement: RA-010-D0RA02455D-s001 [file RA-010-D0RA02455D-s001.pdf]

Electronic Supplementary Material (ESI) for RSC Advances.
This journal is © The Royal Society of Chemistry 2020

Supporting information

An efficient and robust exfoliated bentonite/Ag_3PO_4/AgBr plasmonic photocatalyst for degradation of parabens

Jianchao Ma[a], Shurong Yang[a], Huixian Shi[b,*], Jin Pang[a], Xiaopeng Zhang[a], Yuxing Wang[a], Hongqi Sun[c,*]

[a]College of Mining Engineering, Taiyuan University of Technology, Taiyuan 030024, Shanxi, P.R. China

[b]Institute of New Carbon Materials, Taiyuan University of Technology, Taiyuan 030024, Shanxi, P.R. China

[c]School of Engineering, Edith Cowan University, Joondalup, Western Australia, 6027, Australia

*Corresponding authors.

E-mail: shihuixian@tyut.edu.cn (H. Shi), h.sun@ecu.edu.au (H. Sun)

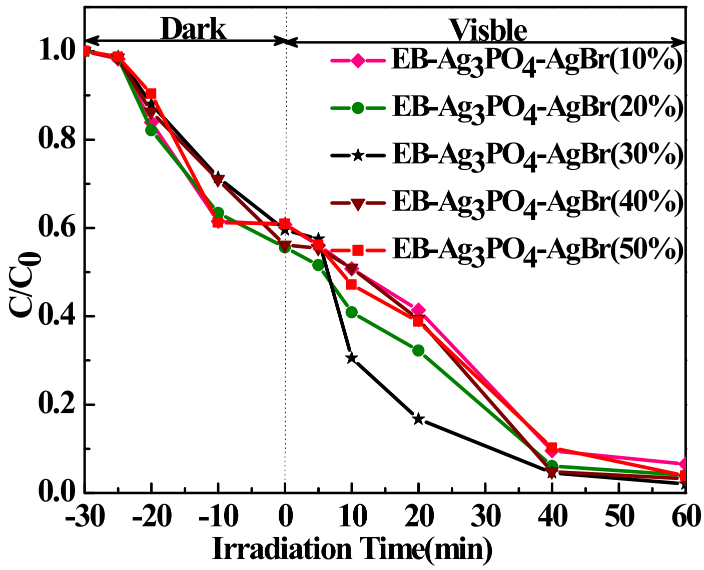

Fig. S1. The effect of the ratio of AgBr to Ag_3PO_4 in different mass ratio on the degradation of MPB.

Table S1. Textural properties of the samples

Exfoliated-bentonite	16.3595	0.022304	43.185
Name	BET/(m^2/g)	Pore volume/(cm^3/g)	Average aperture/(Å)
EB/Ag_3PO_4	4.5035	0.029572	47.788
EB/Ag_3PO_4/AgBr	13.2130	0.057100	100.378

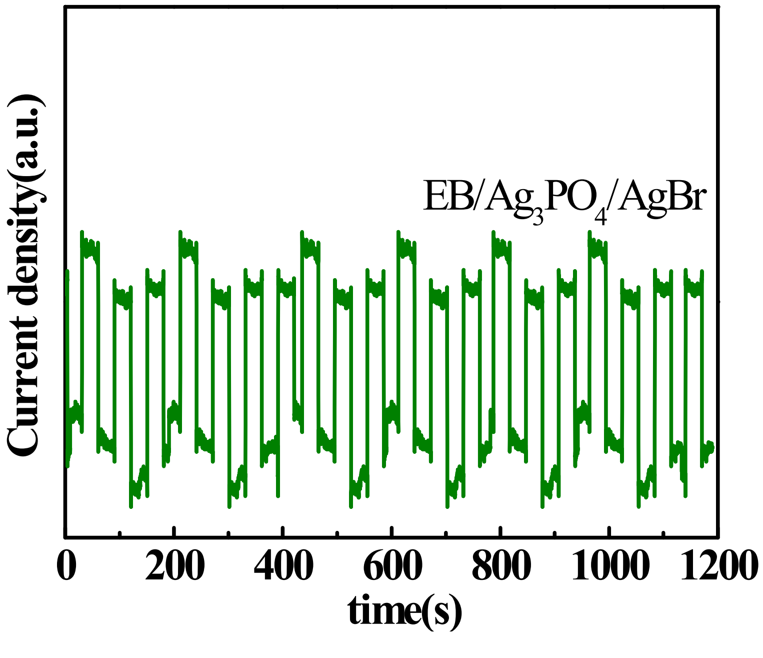

Fig. S2. Transient photocurrent response for the EB/Ag_3PO_4/AgBr (30%) hybrids under visible light irradiation;

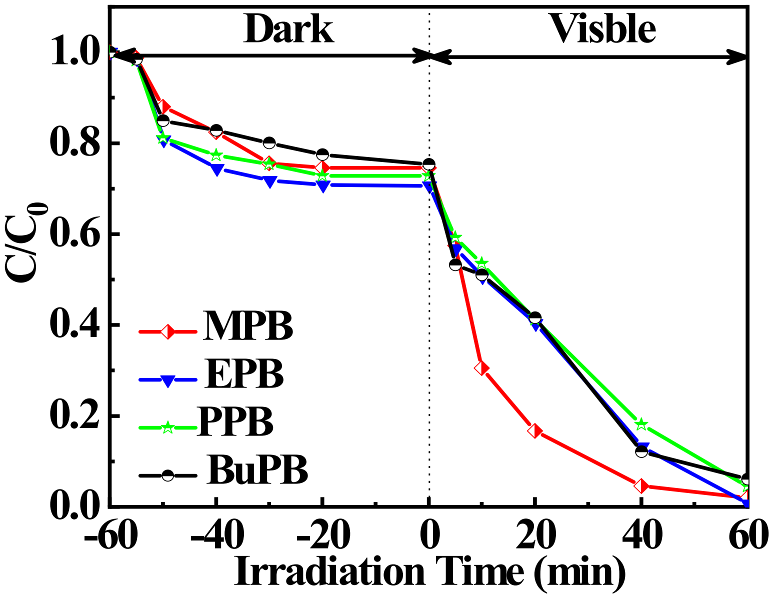

Fig. S3 Relationship curves between irradiation time and C/C_0 for MBP, EPB, PPB and BuPB on $EB/Ag_3PO_4/AgBr$ (30%) composites.

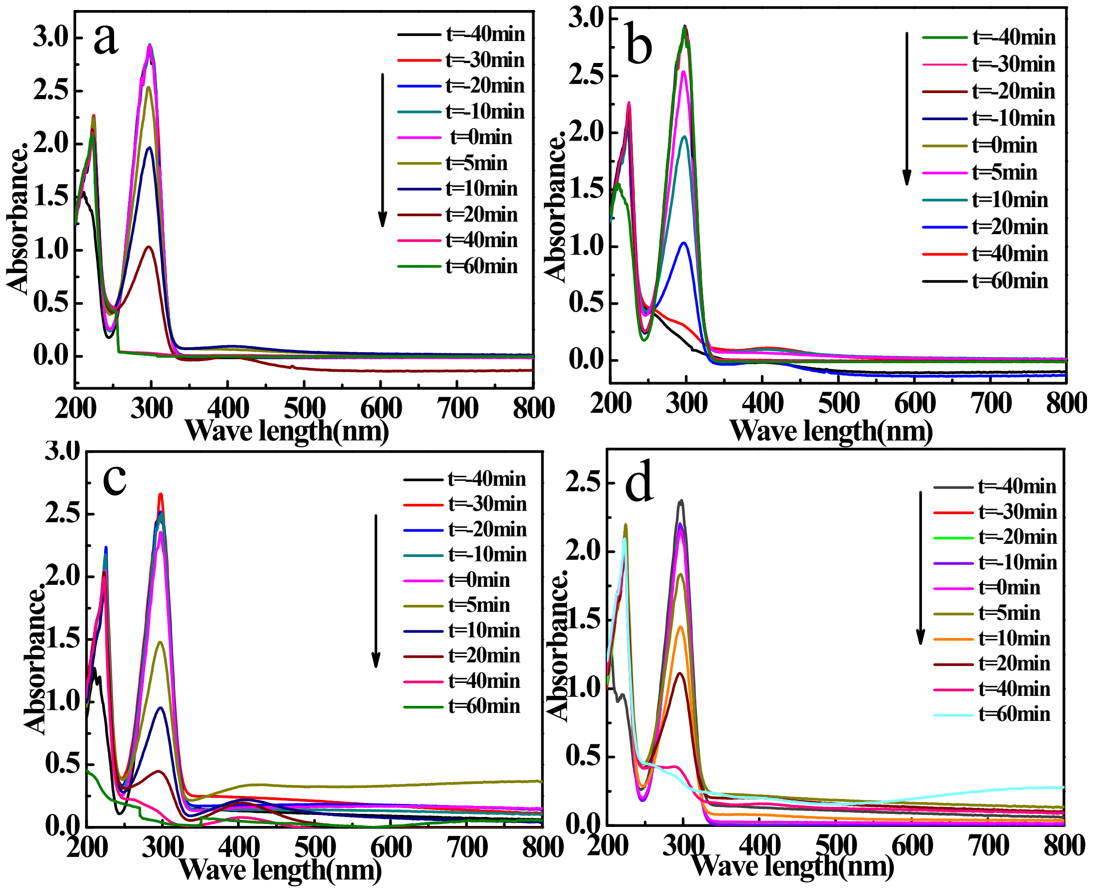

Fig. S4. UV-vis spectra changes of (a) MPB，(b) EPB，(c) PPB，(d) BuPB solution during the photocatalytic degradation by the as-prepared photocatalyst in visible light illumination

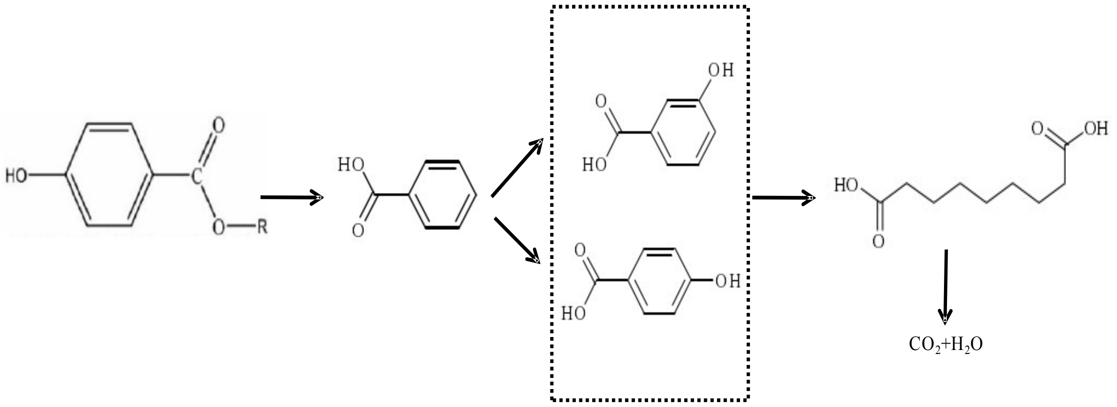

Fig. S5. Photodegradation pathways of methylparaben
